# Design and validation of a novel online platform to support the usability evaluation of wearable robotic devices

**DOI:** 10.1017/wtc.2022.31

**Published:** 2023-01-24

**Authors:** Jan T. Meyer, Natalie Tanczak, Christoph M. Kanzler, Colin Pelletier, Roger Gassert, Olivier Lambercy

**Affiliations:** 1 Rehabilitation Engineering Laboratory, Department of Health Sciences and Technology, ETH Zürich, Zürich, Switzerland; 2 Future Health Technologies, Singapore-ETH Centre, Campus for Research Excellence and Technological Enterprise (CREATE), Singapore, Singapore

**Keywords:** usability evaluation, user-centered design, wearable robotic devices

## Abstract

Wearable robotic devices (WRD) are still struggling to fulfill their vast potential. Inadequate daily life usability is one of the main hindrances to increased technology acceptance. Improving usability evaluation practices during the development of WRD could help address these limitations. In this work, we present the design and validation of a novel online platform aiming to fill this gap, the Interactive Usability Toolbox (IUT). This platform consists of a public website that offers an interactive, context-specific search within a database of 154 user research methods and educational information about usability. In a dedicated study, the effect of this platform to support usability evaluation was investigated. Twelve WRD experts were asked to complete the task of defining usability evaluation protocols for two specific use cases. The platform was provided to support one of the use cases. The quality and composition of the proposed protocols were assessed by (i) two blinded reviewers, (ii) the participants themselves, and (iii) the study coordinators. We showed that using the IUT significantly affected the proposed evaluation focus, shifting protocols from mainly effectiveness-oriented to more user-focused studies. The protocol quality, as rated by the external reviewers, remained equivalent to those designed with conventional strategies. A mixed-method usability evaluation of the platform yielded an overall positive image, with detailed suggestions for further improvements. The IUT is expected to positively affect the evaluation and development of WRD through its educational value, the context-specific recommendations supporting ongoing benchmarking endeavors, and highlighting the value of qualitative user research.

## 1. Introduction

Wearable robotic devices (WRD) are becoming increasingly popular as technologies to enhance human movement and physical abilities. As a whole, WRD provide the potential to assist motion, support neurorehabilitation therapy, or augment existing functionalities (Pons, 2008). These devices can come in the form of wearable exoskeletons, prosthetics, or orthoses and range from fully mobile to stationary systems. As of today, few WRD can be found commercially, with wearable robotic orthoses to assist overground walking, or devices assisting workers in physically demanding tasks in industrial applications being two of the most advanced and marketed use cases. Although substantial technological advancements in the past decades have brought forward more compliant, lightweight, and compact solutions, the currently available devices on the market struggle to meet the large user and market needs (Crea et al., 2021; Xiloyannis et al., 2021). Often, inadequate usability in the dynamic daily life applications appears to be one of the leading causes of the slow adoption and prevalent technology acceptance issues of WRD (Abouhossein et al., 2018; Babič et al., 2021). The limited usability of WRD likely results from the overwhelming complexity of devices. There appears to be a persistent need to simplify the designs of WRD, while ensuring that they successfully meet the target user needs (Arthanat et al., 2007; Hill et al., 2017; Crea et al., 2021; Kermavnar et al., 2021).

To address these issues, a range of studies have shown the value of involving target users in the design of WRD by considering and evaluating their needs and wishes through a systematic, iterative user-centered design (UCD) process (Almenara et al., 2017; Power et al., 2018; Hensel and Keil, 2019; Meyer et al., 2019). A key element in UCD are usability evaluations, which are essential to assess whether the WRD fulfills the needs of the user and to identify potential issues which may need to be addressed in another design iteration (Poulson and Richardson, 1998; Christ et al., 2012; Davis et al., 2020).

Even though standardized definitions and methodologies for usability evaluation exist, the understanding of the term as well as the daily practice varies greatly within the WRD field. This creates difficulties in adequately evaluating usability, defining what a high quality usability evaluation would look like, as well as comparing results to similar contexts of use (Meyer et al., 2021). The International Organization for Standardization (ISO) defines usability as the “extent to which the user’s physical, cognitive and emotional responses that result from the use of a system, product, or service meet the user’s needs and expectation” (International Organization for Standardization, 2018). From the perspective of this definition, it becomes apparent that the majority of WRD studies focus mainly on the effectiveness dimension of usability. The effectiveness of the WRD has been investigated intensively, mainly by employing performance metrics (task success, time for task), kinetic and kinematic analyses, or physiological measurements in efforts to quantify the positive effects of WRD usage (Pinto-Fernandez et al., 2020; Crea et al., 2021; Kermavnar et al., 2021). Unfortunately, such evidence generated under controlled, in-lab testing conditions seldom transfers to real-life applications, as the usage of these devices outside of these controlled environments is dynamic and unpredictable. Thus, there is a need to precisely define the specific context of use when selecting evaluation protocols, before then iteratively evaluating WRD throughout their technological maturation. This goes from basic idea all the way to a commercialized product, while continuing to compare their usability throughout. In addition, the quantitative data are rarely validated or supplemented with qualitative data which would likely better reflect target users’ opinions. Despite the value of mixed-method approaches to UCD and usability evaluation practice (Sauro, 2011; Albert and Tullis, 2013), there are significantly less reported qualitative data in the scientific literature of WRD evaluation (Torricelli et al., 2020; Meyer et al., 2021). Beyond this, the majority of WRD studies are also limited in their comparability and generalizability, as non-validated and non-standardized measures are predominantly used (Ármannsdóttir et al., 2020). Ultimately, a methodological change is needed to create a benchmarking ecosystem and improved practice. With this, the quality and comparability of WRD usability evaluations could be increased to address the prevalent technology acceptance limitations (Torricelli et al., 2020; Babič et al., 2021).

Habitually, the task of defining WRD evaluation protocols is initiated by searching for state-of-the-art scientific literature. Outcome measures and study protocols are then identified from the published pool of studies. This appears to be the first issue of WRD evaluation, as the published materials seldom show all data (such as qualitative analyses) relevant to the WRD design and often lack good practices such as the use of validated tools. Besides specific WRD evaluation literature, platforms such as the Rehabilitation Measures Database from the Shirley Ryan Ability Lab^1^ or the COVR toolkit to assess robotics safety^2^ could be used to find relevant outcome measures. Furthermore, general toolkits, websites, and books that provide information on usability evaluation methods could be used to provide an overview of user research methods (Hom, 1998; Rubin and Chisnell, 2008; Wilson, 2013; Sauro and Lewis, 2016). Despite these existing resources, WRD developers have repeatedly reported that the search for relevant and context-specific methods is a key limiting factor to their evaluation practice (Meyer et al., 2021). As such, it appears that dedicated resources to find context-specific information on usability evaluation to support a user-centered decision-making are still missing.

In this work, we present the design and evaluation of an open-access platform for wearable robotics developers – the Interactive Usability Toolbox (IUT). It aims to support the user-centered evaluation of WRD by facilitating the search and selection of context-specific outcome measures. The platform consists of a public website that offers an interactive search in an online database of 154 user research methods. Additionally, the tool aims to serve an educational purpose to allow WRD developers to familiarize themselves with usability and user research methods to make more informed decisions regarding the design and evaluation of their work. We hypothesize that we can positively influence the usability evaluation practice of WRD by supporting developers in understanding usability and creating more user-focused, mixed-method evaluation protocols. For this, we asked 12 WRD experts to define usability evaluation protocols for 2 fictional use cases, once with and once without the IUT. Aiming for an unbiased evaluation, the content and quality of the protocols were assessed by the (i) two blinded reviewers, (ii) the participants themselves, and (iii) an analysis of the protocols performed by the study coordinators. Ultimately, by providing an online platform to find and select usability evaluation measures, we look to support developers in increasing the usability of WRD across the whole field.

## 2. Design of the IUT

### 2.1. Back end

First, a database of usability evaluation methods and measures, hereon referred to as items, was first established. General usability testing methods were identified from established literature in user experience research (Rubin and Chisnell, 2008; Albert and Tullis, 2013; Wilson, 2013). Clinical measures which may be used to investigate the usability of WRD for medical applications were derived from existing databases such as the Rehabilitation Measures Database from the Shirley Ryan Ability Lab^3^ as well as the Physiopedia website.^4^ In combination with an extensive literature research, as well as a dedicated survey on WRD usability evaluation practices launched in 2020 (which included 158 responses from WRD developers), all data on evaluation metrics for WRD were compiled into a database containing 154 usability evaluation items (Meyer et al., 2021). The items in the database range from generic research methods (*user interview* or *think-aloud*) to specific measures such as validated questionnaires (*System Usability Scale*) or clinical tests (*Timed Up and Go Test*).

With a wide range of items in the database, it was essential to find a way to classify their suitability for specific WRD contexts of use. For example, some items may be specifically designed for people with gait impairments after stroke and thus are only relevant for WRD that target this population and use case. Therefore, each item was characterized by the following context of use specifications: 1) target population, 2) target age group, 3) target body area, 4) fit for technology readiness level (TRL), 5) evaluation perspective (observed, user, expert), and 6) general data method as defined by the ISO TR 16982 (performance-related measurement, survey/questionnaires, etc.) (International Organization for Standardization, 2002). Generic evaluation items were characterized to cover all possible selections of each specification, whereas specific items were limited in their scope and fit accordingly. A short description and an additional external source for more information were provided for each item.

While this classification allows the IUT to filter the items for a defined context of use, a more specific link to the item’s evaluation scope in terms of usability had to be established. Although the ISO aims to provide a clear definition to the term usability (International Organization for Standardization, 2018), WRD developers seldom use the three dedicated dimensions *effectiveness*, *efficiency*, and *satisfaction* to describe user experience of human–robot interactions. In our previous works investigating usability evaluation practices in wearable robotics, we identified that usability attributes such as *functionality*, *ease of use*, or *comfort* are most frequently used to describe usability (Meyer et al., 2021; Gantenbein et al., 2022). These insights were combined with dedicated works investigating usability terminologies and practices (Hornbæk, 2006), to form a long-list of 48 individual usability attributes. In a dedicated working group with eight WRD stakeholders, the link between these attributes and the three main usability dimensions were discussed. Each participant of the working group independently rated the link of each attribute to the dimensions *effectiveness*, *efficiency*, and *satisfaction* (1 = not linked at all, 10 = strongly linked). The median values across the working group were then established as the “usability focus” of each usability attribute. For example, the focus of attribute *functionality* was established as: *effectiveness* = 8.0, *efficiency* = 5.0, and *satisfaction* = 5.0. The complete list of attributes and their usability focus is provided in the Supplementary Materials. As a last step, each evaluation item was assigned a range of usability attributes that either are specifically or potentially evaluated with the evaluation item. The assignments of attributes were done by the study coordinators and were based on data from outcome measure validation studies where available. For those items where no such data were available, the consortium of authors discussed until consensus was found. The individual assignments of attributes for each evaluation item can be found on the IUT website.

### 2.2. Front end

Once the back-end was established, a user interface was designed that allows WRD developers to browse the established database and find context-specific evaluation items. For this, an open access platform – the IUT – accessible via a public website https://www.usabilitytoolbox.ch/ was developed. A range of functions were integrated to allow the user to make informed decisions on how and why to choose specific evaluation items. The tools developed as part of this work were (i) the “Wizard” tool, (ii) the direct database search, and (iii) the usability wiki page.

**Figure 1.**
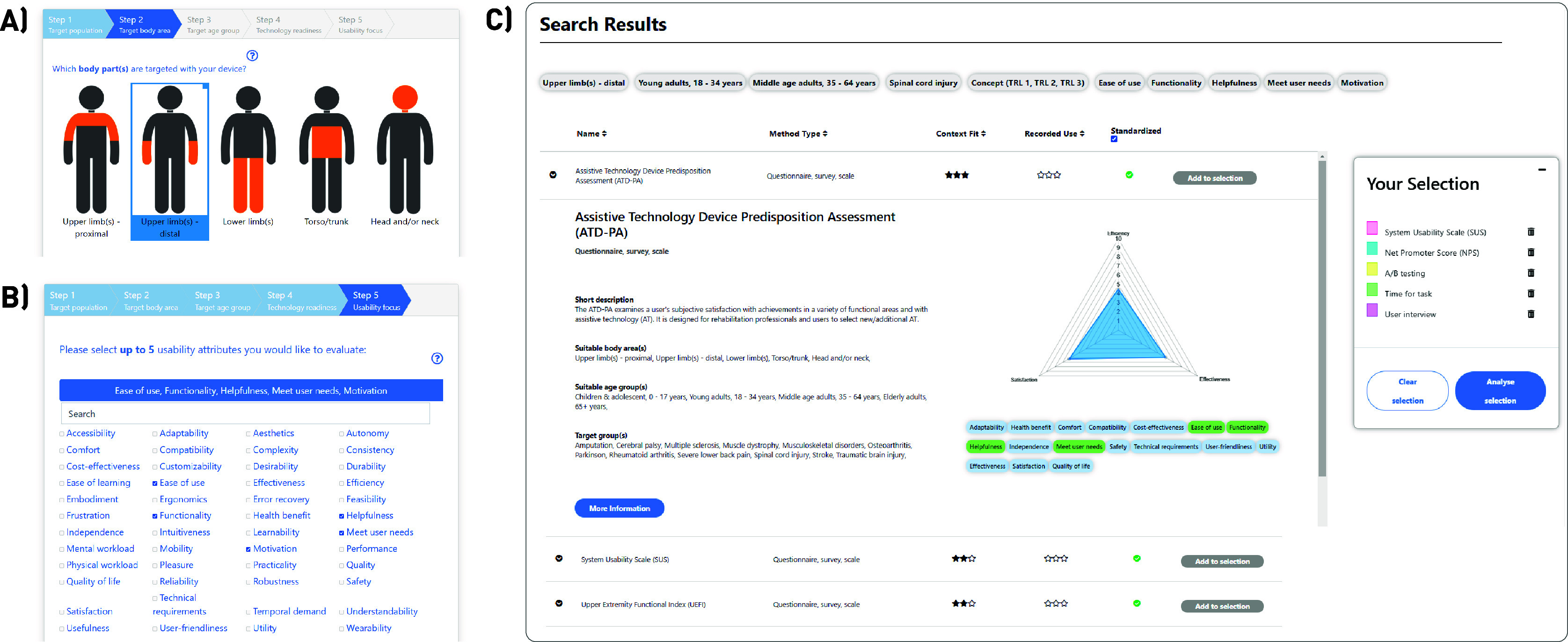
Overview of the Wizard tool of the Interactive Usability Toolbox: (a) Interface of the Wizard, with a step-by-step guide in defining the WRD context of use, (b) The last step of the Wizard is the definition of the evaluation focus by selecting a maximum of five usability attributes, (c) Result of the Wizard search, with a list of evaluation items listed by their Context Fit and Recorded Use. Individual items can be added to a selection and later viewed in bulk in a summary page.

The Wizard tool guides the user in defining the context of use of a WRD for which the usability is to be evaluated. Step by step, the following information can be entered: (1) general usage purpose (augmentation, assistance, or therapy) and target population, (2) target body area, (3) targeted age group, and (4) TRL. Figure 1(a) shows the basic interface of the Wizard in the example of step 2. In the last step of the Wizard (step 5), the usability focus can be specified with the 48 provided usability attributes (Figure 1(b)). To make the search as specific as possible, the Wizard only allows to select a maximum of five attributes per search. After submitting the context-specific search, the IUT opens a new tab displaying the search results as shown in Figure 1(c).

The items displayed in the search results are then listed according to their “Context Fit,” a 4-level scale (zero to three stars) of how well an evaluation item fits the entered context of use. Only items that fit at least one of the context of use information entered in steps 1–5 are displayed. Three stars (maximum) are attributed to evaluation items that cover at least 80% of the context of use specifications. The Context Fit allows the user to find context-specific measures but does not provide any information about the relevance of the evaluation item for the WRD field. To complement this, the “Recorded Use” rating (same star rating system) was established. The Recorded Use displays if and to what extent a measure has previously been used in the specifically entered context of use (see Figure 1(c)). The data for these ratings were generated from the results of our previously published survey on usability evaluation practices of 158 WRD developers, which contained the same pieces of context of use information (Meyer et al., 2021). The IUT thus aims to facilitate the search for usability evaluation items through the interactive recommendation list, ordering items by their Context Fit, their Recorded Use, as well as characterization details and educational information.

## 3. Evaluation of IUT validity and usability

### 3.1. Study design

**Figure 2.**
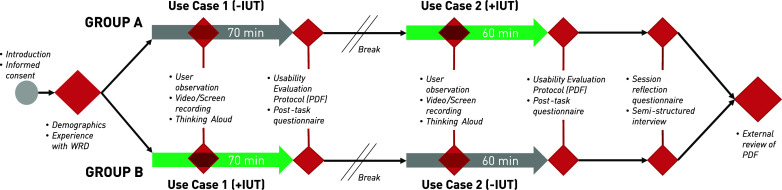
Overview of the study design: A cross-over study design was applied, with the Interactive Usability Toolbox as intervention (IUT, green color). Participants were randomly allocated to Group A or B, either starting with (+IUT) or without (−IUT) the toolbox. All data collection points are marked in red, and the outcome measures applied during the data collection points listed as bullet points.

A single-session, cross-over study was conducted to investigate the influence of the IUT as a novel online platform to facilitate WRD usability evaluation (see Figure 2). The main task of the study was to prepare usability evaluation protocols for two defined, fictional WRD use cases. The IUT was provided as an additional resource to complete one of the two tasks. The effect on the quality and content of the evaluation protocols was investigated. In short, the two uses cases addressed were:

- *Use Case 1 (UC1):* Passive, overhead exoskeleton for the automotive industry. Technology maturity state: Design concepts formulated, no functional prototype available.
- *Use Case 2 (UC2):* Active, ankle-foot orthosis for people with gait deficits. Technology maturity state: Prototype safety previously tested with neurologically intact volunteers.

These descriptions of the use cases are simplified. More detailed formulations were provided to the participants. The ad-verbum descriptions of the use cases can be found in the Supplementary Materials. All participants completed the same task twice, once for each use case. The UC1 was always completed first, followed by UC2. The participants were randomly assigned to two groups: Group A (*n* = 6) completed UC1 without the IUT (−IUT) and UC2 with the IUT (+IUT). Group B (*n* = 6) completed UC1 with the IUT (+IUT) and UC2 without IUT (−IUT). In the +IUT condition, a link to the IUT was provided and the participants were informed that the platform could be used to support the task. In the −IUT condition, the participants were asked to approach the task with their conventional strategy and were permitted to use any resource except the IUT. A cross-over design was chosen to ensure that both use cases were tested with and without the IUT. This also ensured that the effect of the IUT could be evaluated independently of the specific use case. This further allowed both groups to serve as opposite controls within the two use cases, alternating whether they started with or without the IUT. The time given for the task was 70 min for UC1 and 60 min for UC2. Given the task complexity, 10 min was added to the first task for familiarization. A usability evaluation protocol template was provided to the participants, highlighting the minimally expected details to be defined. These included the study aim, target population, study design, and outcome measures (see the Supplementary Materials for more detail). Once the task was completed, a PDF of the protocol was created and saved as the task output.

### 3.2. Sample

The participant sample of WRD experts was recruited from the authors’ wider network and contacted via email or social media. Inclusion criteria consisted of (1) minimum 36 months of experience in wearable robotics research and development and (2) aged 18 or above. Exclusion criteria consisted of (1) prior experience and familiarity with the IUT and (2) severe aphasia or cognitive impairments which would prevent them from following study instructions. Eligible candidates completed a wearable robotics experience questionnaire before participating in the study to validate their eligibility. The participants were asked to estimate their level of experience in UCD and usability testing by estimating the number of dedicated usability studies they have conducted and the number of target users they have interacted with. After completing each task, participants were asked to rate their familiarity (1 = very unfamiliar, 10 = very familiar) and level of evaluation expertise (1 = very unqualified, 10 = very qualified) for the presented use case. An overall use case expertise score was calculated by taking the product of both values. A heterogeneous sample (across all participants) was targeted in terms of levels of expertise and backgrounds. Building on the usability evaluation principle established by Nielsen and Landauer (1993), six participants per group were assumed to be sufficient to adequately test the basic validity of the IUT approach and to identify many residual usability problems that may occur with the IUT. The targeted sample size was, therefore, six participants per group.

### 3.3. Outcome measures

The effect of the IUT on the quality and content of usability evaluation protocols was investigated. To do so, it was necessary to identify criteria and measures to assess usability evaluation protocols quality. The understanding of protocol quality likely differs depending on the perspective and expertise. Therefore, we investigated three different perspectives: (i) the view of two external, unbiased, and blinded reviewers, (ii) the perspectives of the participants themselves, and (iii) the perspective of the study coordinators.

#### 3.3.1. External, blinded perspective

To get an external, unbiased, and blinded review of the established protocols, two expert reviewers were recruited. The reviewers were selected based on their specific expertise for the defined use cases. Both reviewers have substantial academic and industrial experience in the dedicated WRD area and have personally worked with products in the niche of their use cases. Before commencing data collection, a grading sheet was developed on the basis of quality criteria that were proposed by the reviewers, allowing them to objectively evaluate the quality of usability evaluation protocols. Twelve anonymized usability evaluation protocols were then provided to each reviewer. For each protocol, an overall rating of quality was assigned (1 = very poor quality, 10 = very high quality), and the 12 protocols were ranked from 1 (best) to 12 (worst). The same grading sheet was used for the two use cases. The grading sheet template can be found in the Supplementary Materials. The reviewers received all 12 protocols for their use case in one batch, in a randomized order. The reviewers were blinded to the condition, as all indicating information was redacted from the protocols. The study coordinators then analyzed the rankings and overall quality grading.

#### 3.3.2. Participant perspective

A list of questionnaires and outcome measures was used to investigate the participants’ perspective of the quality of their own protocols (see Figure 2). After each task, participants were asked to reflect on their protocols through a post-task questionnaire (PTQ). The PTQ comprised of a mix of different scales and questions. At first, the Single-Ease-Question (SEQ), a standardized measure of task difficulty, was rated (Sauro and Lewis, 2016). Then, a set of custom-made questions (11-point Likert scales) gauged their own perception of the protocol quality. Finally, the participants were asked to reflect on their protocol focus by allocating a total of 100 points across the three usability dimensions: *effectiveness*, *satisfaction*, and *efficiency.* Standardized definitions of each of these usability dimensions were accessible via a help function in the evaluation questionnaire. After completing the second task, participants were asked to complete a session reflection questionnaire (SRQ) which allowed them to compare their task experiences. In the SRQ, participants stated their level of agreement on a 5-point Likert scale (fully disagree to fully agree) to a range of statements aiming to investigate and distinguish the potential learning effects from either sheer task repetition or the new knowledge and expertise gained from the IUT.

#### 3.3.3. Study coordinator perspective

In previous works by the study coordinators (Meyer et al., 2021), common quality limitations of WRD studies were identified as:

- Device-oriented studies with an inadequate balance of evaluation focus between the usability dimensions *effectiveness*, *satisfaction*, and *efficiency.*
- Insufficient usage and reporting of qualitative data to support quantitative findings and reflect user opinions.
- Insufficient generalization and comparability of evaluation results due to limited use of standardized, validated measures in favor of customized tools.

From the perspective of the study coordinators, an effect of the IUT that would help addressing these limitations would be considered as an improvement of usability evaluation quality. Therefore, the evaluation protocols were analyzed according to these quality criteria. To do so, each protocol was individually screened and analyzed by the study coordinators. All listed evaluation items were categorized and counted. The items were categorized according to the method type (quantitative or qualitative), data type (subjective or objective), and classified as standardized or non-standardized. Unspecific outcome measures were grouped into larger, common item categories, while specific items were directly categorized. For example, if a participant listed “metabolics” or “EMG,” items were grouped as “physiological measures” and categorized as quantitative, objective, and non-standardized. An item such as “System Usability Scale” was directly categorized as quantitative, subjective, and standardized.

### 3.4. Mixed-method usability evaluation of the IUT

After completing both tasks, the participants were asked to rate the usability of the IUT with a range of standardized as well as custom scales. With the Net Promoter Score (NPS), participants rated the question: “How likely would you be to recommend the IUT to a friend or colleague?” from 0 = not likely at all to 10 = extremely likely. The NPS is an established tool for user loyalty and usability, with benchmark scores provided for all kinds of applications (Sauro and Lewis, 2016). The NPS ratings are grouped into promoters (ratings = 9 or 10), passives (ratings = 7 or 8), and detractors (ratings of 6 or lower). To derive the NPS score, the relative number of detractors is subtracted from the relative number of promoters. Additionally, the Post-Study System Usability Questionnaire (PSSUQ) was employed to more specifically assess the IUT website. Within 16 items, rated on a 7-point Likert scale (+N/A option), the PSSUQ assesses System Use (items 1–6), Information Quality (items 7–12), and Interface Quality (items 13–16) (Sauro and Lewis, 2016). To complement the two standardized, general usability measures, a set of custom-made scales (7-point Likert scale) were used to assess the usefulness and desirability of the IUT.

Qualitative data were collected in combination with the quantitative approaches taken. Throughout the sessions, participants were instructed to think out loud, sharing their thoughts as they completed the task. In addition, the participant’s screens were observed by the study coordinator, and notes were taken on their task strategies and interactions with the IUT. At the end of the session (after completing the SRQ), a semi-structured interview was conducted to discuss the task experience and improvement suggestions for the IUT.

### 3.5. Data collection and analysis

All data were collected remotely, and participants were asked to join a virtual meeting on Zoom (Zoom Video Communications, San Jose, CA, USA) via their regular working station (PC, laptop, etc.). Prior to data collection, informed consent and media consent were obtained. The participants were asked to share their screens, and the session was recorded for further data analysis. The task and use case descriptions, and all questionnaires were administered using the QuestionPro Survey Software (QuestionPro Inc., Austin, TX, USA). The raw questionnaire data were exported to MS Excel 365 (Microsoft Corp., Redmond, WA, USA) for post-processing, and statistical analyses were done in RStudio Team 2021 (RStudio PBC, Boston, MA, USA). Two-sample t-tests were applied at a 0.05 significance level under unequal variance assumptions for normally distributed data. The exact Wilcoxon–Mann–Whitney test was applied if a Shapiro–Wilk normality test indicated that normal distribution could not be assumed. In addition, a multiple pairwise comparison was used to investigate individual and interaction effects of the IUT conditions and the UC-specific experience scores on the protocol quality. Individual anecdotes from the think-aloud protocol and semi-structured interviews with participants were reported.

## 4. Results

### 4.1. Participants

In total, 12 participants aged 33.2 (4.2), reported as mean (standard deviation), completed the study protocol. The participants had, on average, 6.6 (1.5) years of experience within the field. The individual number of usability studies conducted and number of user interactions are displayed in Table 1. The mean experience scores for each use case were similar for both study Groups A (+IUT = 29.3, −IUT = 46.0) and B (+IUT = 29.5, −IUT = 38.0).

**Table 1.** Participant demographics and wearable robotic device experience (*n* = 12)

	P1	P2	P3	P4	P5	P6	P7	P8	P9	P10	P11	P12
Years of experience	4.5	6	5	7	6	8	8	7	5.5	6	6	10
Number of usability studies	1	0	1	>100^a^	4	2	1	4	0	3	1	6
Number of users tested with	5	5	11	>1,000^a^	2	6	10	100	10	45	15	>100^a^
Score of expertise (1–100)												
Use Case 1	2	16	48	72	18	36	6	20	45	1	9	80
Use Case 2	49	40	36	25	32	48	64	81	15	2	64	48
Study group	B	B	A	B	A	A	B	A	A	B	A	B

a Numbers were estimated and rounded.

### 4.2. IUT validation

#### 4.2.1. External reviewer perspective

Table 2 shows the quality ratings and the ranking of the usability evaluation protocols of UC1 and UC2. The mean quality score (1 = lowest, 10 = highest) across both use cases (*n* = 24) was 6.42 (2.54) for +IUT condition and 5.50 (2.28) for −IUT. The average rank (1 = best, 12 = worst) of protocols generated in the +IUT and −IUT condition was 5.83 (3.64) and 7.17 (3.43), respectively. Even though a slightly higher mean rank of the protocols generated with the IUT can be observed (especially for Use Case 1), differences in means were insignificant (exact Wilcoxon–Mann–Whitney test, *p* = .39). A multiple pairwise comparison yielded that neither the IUT condition (*p* = .36) nor the experience score (*p* = .96) significantly affected the protocol quality. Also, no significant interaction effect between the condition and experience was observed (*p* = .15).

**Table 2.** External reviewer quality grading (1 = lowest, 10 = highest) and ranking (1–12) of usability evaluation protocols (*n* = 24)

	Use Case 1 (*n* = 12)			Use Case 2 (*n* = 12)		
Rank	Quality rating	Condition	Experience score	Quality rating	Condition	Experience score
1	10	+IUT	1	8	+IUT	48
2	9	−IUT	45	8	−IUT	49
3	9	+IUT	6	7	+IUT	64
4	8	+IUT	72	7	−IUT	48
5	8	+IUT	2	7	−IUT	64
6	8	+IUT	80	5	−IUT	2
7	7	−IUT	48	5	+IUT	15
8	6	−IUT	9	4	+IUT	32
9	6	−IUT	18	3	−IUT	25
10	5	+IUT	16	3	+IUT	81
11	3	−IUT	20	3	−IUT	40
12	2	−IUT	36	2	+IUT	36

#### 4.2.2. Participant perspective

The results from the PTQ in which the participants rated their protocols by a list of quality criteria (rated from 1 = low to 10 = high) are presented in Table 3. The effect of the IUT appears to differ between UC1 and UC2. While the mean ratings of the quality criteria were all rated higher for the +IUT condition when applied to UC2, the opposite was observed for UC1. A low mean rating of the perceived comparability of results was observed for the group that completed UC1 with the IUT. For UC2, the participants that completed the task with the IUT were more confident in their selection of measures, the meaningfulness of the outcomes generated, and the comparability of results. However, none of the use case internal rating differences were found to be statistically significant (*p* > .05). Independent of the condition, all participants agreed that additional time to complete the task would have further improved the quality and content of the protocols. The mean SEQ ratings (1 = very difficult, 4 = neutral, 7 = very easy) indicated that the task was generally perceived as difficult across all participants and conditions (3.75 (1.19)). The only participant subgroup which reported a mean rating above 4 (neutral) was Group A which completed UC2 with the IUT (4.17 (1.72)). No significant difference in task difficulty was observed between the +IUT and −IUT conditions (*p* > .05).

**Table 3.** Perspectives of participants on quality criteria of usability evaluation protocols (11-point Likert scale, *n* = 12)

	Use Case 1 (*n* = 12)		Use Case 2 (*n* = 12)	
	−IUT	+IUT	−IUT	+IUT
A) How satisfied are you with your choices of evaluation measures?	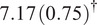	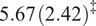	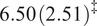	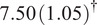
B) How confident are you that this protocol would generate meaningful usability insights?	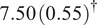	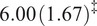	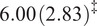	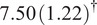
C) How comparable would the data from this study be to existing benchmarks or state-of-the-art studies?	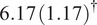	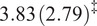	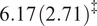	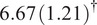
D) How likely would your protocol improve if you would have more time for the task?	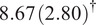	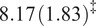	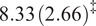	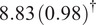

*Note.* Group A ( 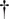 ) and Group B ( 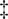 ) are indicated to simplify visual intra-group comparison.

**Figure 3.**
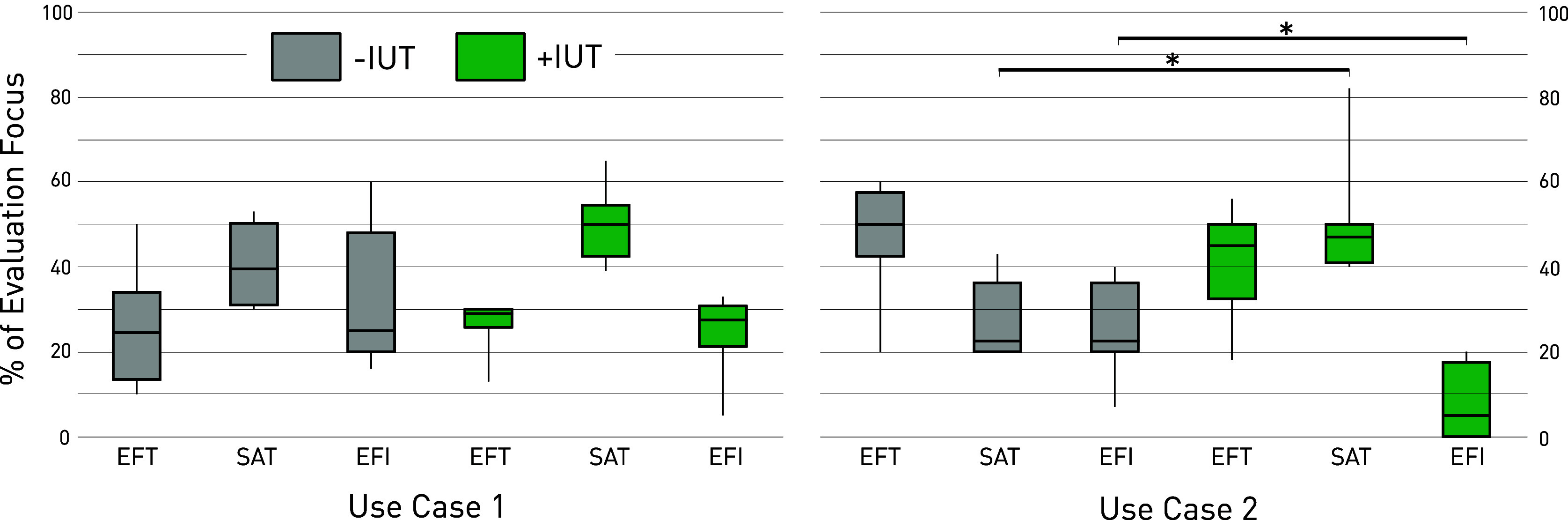
Participant’s view on the usability evaluation focus: The focus of the proposed protocol was rated by the participants by allocating a total of 100 points between the three usability dimensions *effectiveness* (EFT), *satisfaction* (SAT), and *efficiency* (EFI), * = *p* < .05.

The results from the evaluation focus estimation with regard to the three usability dimensions *effectiveness* (EFT), *satisfaction* (SAT), and *efficiency* (EFI) are shown in Figure 3. For UC1, no significant difference was found in evaluation focus both with and without the IUT. In both conditions of UC1, the largest focus was directed toward satisfaction (+IUT; median = 39.5, −IUT; median = 50.0). On the other hand, in UC2, a significant effect of the IUT on the evaluation focus was found. Compared to the −IUT condition, protocols generated with the IUT showed an increased focus on satisfaction (*p* = .016), matched with a decreased focus on efficiency (*p* = .029).

**Figure 4.**
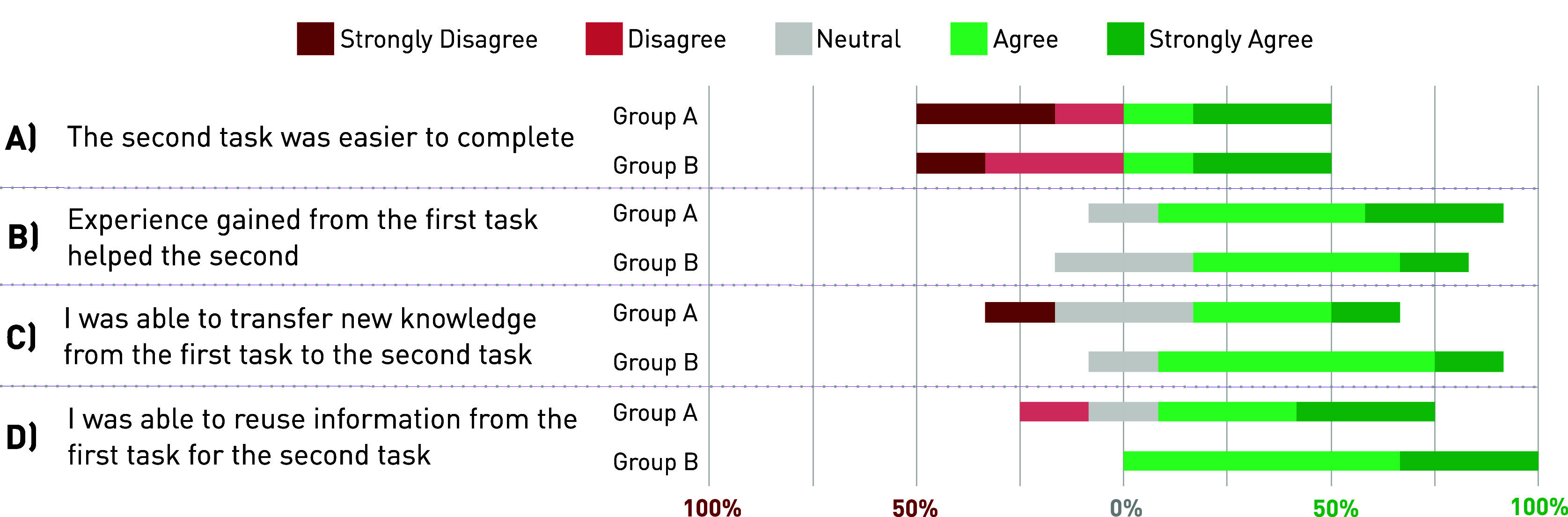
Likert scale results on learning effects and knowledge transfer: The participants of Group A (UC1; −IUT, UC2; +IUT) and Group B (UC1; +IUT, UC2; −IUT) rated their level of agreement to four statements on the knowledge transfer and learning effects.

The participants’ level of agreement with specific statements about learning effects and knowledge transfers is shown in Figure 4. The difference in agreement levels of Group A (UC1; −IUT, UC2; +IUT) and Group B (UC1; +IUT, UC2; −IUT) for each statement was investigated. The levels of agreement and disagreement for statement A, investigating the effect of task repetition on task difficulty, were a 50/50 split in both groups. Both groups showed a high level of agreement with statement B, indicating that experiences from the first task helped the second task. Differences of opinion were found for statements C and D. Group B largely agreed (83.5%) that new knowledge had been transferred from the first to the second task (statement C), whereas 50% from Group A, rated the item with neutral or disagreeing opinion. All participants (100%) of Group B agreed that they could reuse information from the first task (statement D), compared to only 66.5% within Group A.

#### 4.2.3. Study coordinator perspective

Table 4 shows the composition analysis of the designed usability evaluation protocols based on the listed evaluation items. Relative values and ratios of method type, standardization, and data type were calculated for each use case and condition (4× *n* = 6). For both use cases, the protocols from the +IUT contained more individual evaluation items. The selection of evaluation items was generally similar between UC1 and UC2 in the −IUT groups but differed between the respective +IUT groups. For UC1, protocols generated with the IUT showed a reversed ratio in methods types and data types. The +IUT group selected more qualitative items (53%), while the −IUT group focused on quantitative measures (74%). The standardization ratio in UC1 protocols remained unaffected by the IUT. In contrast, method types remained similar for both conditions in UC2, but more standardized measures were listed through the use of the platform (+IUT = 38%, −IUT = 24%). For both use cases, the IUT appeared to promote the use of subjective evaluation items shifting the respective ratios compared to the −IUT groups.

**Table 4.** Analysis of evaluation protocol composition (*n* = 24)

	Use Case 1 (*n* = 12)		Use Case 2 (*n* = 12)	
	−IUT (*n* = 6)	+IUT (*n* = 6)	−IUT (*n* = 6)	+IUT (*n* = 6)
*Evaluation items*				
Total # of items	34	43	41	53
Average # of items per protocol	5.67	7.17	6.83	8.83
# of individual items	16	20	20	25
*Method type*				
Quantitative item	74%	47%	76%	79%
Qualitative items	26%	53%	24%	21%
Ratio	2.78	0.87	3.10	3.82
*Standardization*				
Non-standardized items	88%	88%	76%	62%
Standardized items	12%	12%	24%	38%
Ratio	7.50	7.60	3.10	1.65
*Data type*				
Objective items	56%	33%	51%	42%
Subjective items	44%	67%	49%	58%
Ratio	1.27	0.48	1.05	0.71

### 4.3. IUT usability

#### 4.3.1. Quantitative evaluation

**Figure 5.**
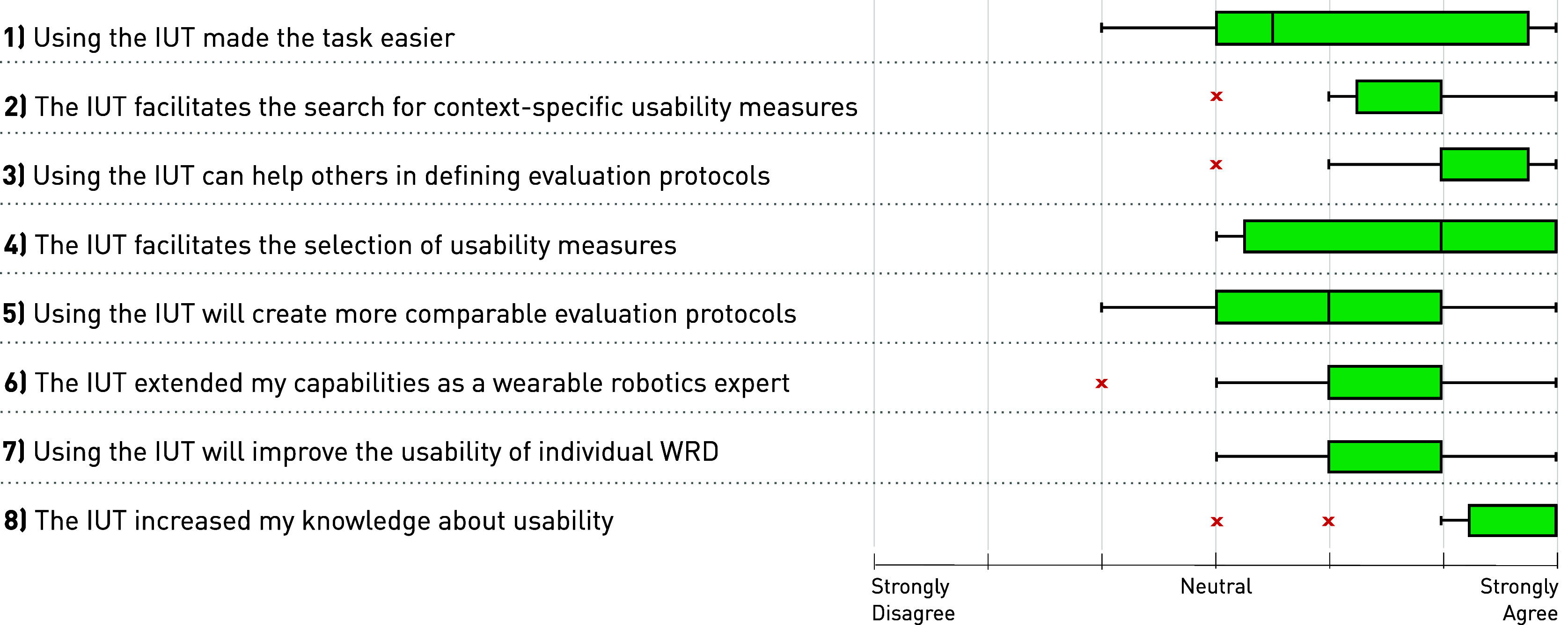
Likert scale results from the custom usability questionnaire: The level of agreement to eight statements about the IUT impact on the study task WRD evaluation was rated from 1 = strongly disagree to 10 = strongly agree.

From the individual NPS ratings, the IUT received a score of +25% (2 promoter, 8 neutral, 2 detractor). Furthermore, a global PSSUQ score of 76.41% was derived from the subscale ratings of System Use = 79.17%, Interface Quality = 77.68%, and Information Quality = 72.82%. The results from the custom Likert scale questionnaire on IUT usability are shown in Figure 5. The level of agreement to all eight statements was rated on a 7-point scale (1 = strongly disagree, 7 = strongly agree). The mean ratings of all eight items were above the “neutral” score (4.00). The highest (most positively) rated items collectively show that the IUT facilitates the search for context-specific measures (item 2 mean rating = 5.86 (0.83)), that it can help defining evaluation protocols (item 3; 6.00 (0.85)), and that it can increase knowledge about usability (item 8; 6.50 (1.00)).

#### 4.3.2. Qualitative evaluation

A wide range of individual statements were recorded from both the think-aloud and the semi-structured interview with each participant. Regarding general IUT desirability, five participants (80%) of Group B remarked during think-aloud that they would have also liked to use the IUT for their respective second task. Similarly, all participants of Group A mentioned that they would have likely benefited from using the IUT in their first task. The desirability and need for a platform like the IUT were a common theme with statements like: *“I felt the need for something like [the IUT] because I am doing a usability study right now. If I would have had the tool before, I probably would have improved the protocol.”*, or *“I think [the IUT] is really good. I found many relevant measures that I was not aware of.”* Several participants remarked that they appreciate such developments that aim at facilitating WRD research in statements such as: *“I really appreciate that someone is developing tools to ease the research life of others. This is a valuable thing to me and should be one of the main aims of research in general: research to improve research quality.”* The importance of usability and the scattered understanding of the term were major discussion points in the semi-structured interviews. Four participants stated that they have not considered attributes such as functionality or performance as being part of usability, but rather that usability and satisfaction are somewhat synonyms. The educational value of the IUT on the concept of usability was mentioned several times, with comments such as: *“[the IUT] was nice to see and spend time on, especially to learn more about usability.* *”* One participant that had rated the NPS with a “detractor” score (=5) later stated in the open discussion: *“thinking about it now, [the IUT] actually expanded my knowledge so now I actually would recommend it.* *”*

Critical feedback and suggestions for improvements were also collected. Specifically, it was remarked that although the IUT supported the search for context-specific evaluation items, it did not sufficiently help with the selection. The provided summary function listed all selected items again in full detail on a new page. Here, participants expected more information in order to make an informed decision on which one(s) to choose: *“[The IUT] could be more [clear on] what the outcome measure exactly does. I felt that that was the information missing to make the tool more complete,”* or *“[the platform] did not really help me select [measures]. I would expect in the future to really help cover the usability triangle of effectiveness, efficiency, and satisfaction.* *”* Two participants recommended creating shortcuts to scientific literature that used the selected evaluation items, to better relate the IUT recommendations to the state-of-the-art studies. Further usability limitations could be identified through user observations. For example, four participants instinctively closed the pop-up window that was designed to showed up when completing the first Wizard search. The pop-up contained a short tutorial explaining the information and interfaces of the Wizard results (see Figure 1(c)). Closing this window prematurely leads to a limited understanding of the provided functions such as filtering options to optimally browse the list of recommended measures. In addition to these comments and suggestions, the intense usability testing provided the means to identify and fix minor website bugs such as empty links or non-functional buttons.

## 5. Discussion

A key limiting factor in the usability evaluation of WRD is the missing availability of resources supporting the selection of outcome measures for specific use cases. In this work, the design and evaluation of a novel platform to support the usability evaluation of WRD were shown. The main features of the IUT, an open-access platform, were introduced, and its mechanisms to find and select context-specific evaluation measures were elaborated. In a dedicated validation study, the influence of the IUT on the design of usability evaluation protocols was investigated. Also, the usability of the IUT website was assessed using a mixed-method approach, and areas of improvement to further optimize the IUT were identified.

### 5.1. Effects on usability evaluation protocol

To better understand the influence of the IUT on usability evaluation protocols, the insights from the three different perspectives about protocol quality have to be combined. From the perspective of the study collaborators, an analysis of the composition of the evaluation protocols revealed that the effect of the IUT on the composition of the usability evaluation protocols differed depending on the use case and was thus context-specific. For UC1 (a design concept), the IUT promoted a mixed-method study design, which follows the objective of the IUT to put more focus on the user perspective as recommended for WRD usability studies (Curry and Nunez-Smith, 2014). Less standardized tools were used for the +IUT condition in UC1, contradicting the proposed aim of the IUT. Nevertheless, one could argue that in the early technology maturity stages, a stronger focus on non-standardized evaluation items such as user interviews or focus groups is more appropriate than standardized tools. Still, the participants who used the IUT for UC1 also realized that they selected less standardized tools and consequently rated their confidence in the protocol and the generalizablity of the resulting data lower than for the −IUT condition. However, for UC2 (a functioning prototype), we observed the opposite effect. In this condition, the IUT promoted the use of standardized tools. Across both use cases, protocols generated in the +IUT conditions included more subjective evaluations, therefore putting more focus on the user satisfaction by collecting data about their personal experiences and opinions. This was reflected not only in the composition analysis but also in the participants own ratings of evaluation focus.

In contrast, as expected, the protocols generated in the −IUT condition showed predominant usage of quantitative, objective, and non-standardized measures. This represents current practices in WRD research, where a high prevalence of custom tools is observed at the expense of standardized alternatives (Koumpouros, 2016; Ármannsdóttir et al., 2020; Meyer et al., 2021). The effectiveness- and device-focused WRD evaluations are a commonly observed practice and a major limiting factor in providing user-oriented usability evidence to address the prevalent acceptance issues of WRD (Pinto-Fernandez et al., 2020; Crea et al., 2021; Xiloyannis et al., 2021). Interestingly, we could also observe that the usability evaluation focus from our participants produced very similar results as found in our recent survey on the usability evaluation practices of 158 WRD developers (Meyer et al., 2021). This, to some extent, validates the dataset from this study which strengthens the argument that the IUT can help WRD developers change their evaluation focus to more user-oriented practices after informing themselves about usability. However, the shift toward more user-oriented evaluations did not make the protocols perfectly balanced across the three usability dimensions (33.3% for each). Instead, the observed increased focus on *satisfaction* came at the expense of items that would otherwise evaluate *efficiency.* It remains difficult to conclude if equal attention to each usability dimension would further increase the quality of WRD evaluation protocols.

The participant’s perspectives and the author’s analysis of the protocols composition clearly showed an effect of the IUT on the design of usability evaluation protocols. Interestingly, this effect did not significantly affect the quality of the protocols as rated from a completely blinded, external perspective. We can thus argue that the IUT helped change the composition of the protocols to put a larger focus on user satisfaction without affecting the quality of the protocols. It is important to state that the selection of more subjective and more standardized measures generally increased the total number of items listed in the evaluation protocols. While this would increase the length of a usability study, we believe that the benefit of more user-oriented data outweighs this drawback. Nevertheless, one should be mindful of the expected workload and duration of usability protocols.

### 5.2. Usability of the IUT platform

The need for better usability evaluation practices in WRD development is apparent. Both the usability and the potential of the IUT to address this need were investigated with a mixed-method approach. Our results suggest that the research and design efforts to develop the IUT were overall appreciated by all study participants, with an overarching positive outlook from different usability metrics. The PSSUQ and NPS ratings suggest that the website design and features were well perceived; however, there is remaining room for improvement to generate a larger promoter base (Sauro and Lewis, 2016). The educational aspect of the IUT was particularly perceived as helpful for the design of usability evaluation protocols. Considering that, on average, the participants had 6.6 years of WRD experience, their experiences with target users and dedicated usability studies were surprisingly low. This falls in line with the generally low number of (published) usability studies in WRD practice (Reinkensmeyer, 2019). However, these numbers may have to be interpreted with caution. In the post-session interview, it was revealed that many experts under-reported the true number of usability evaluations they have conducted as part of their device evaluation due to different interpretations of what “dedicated usability study” means. Most WRD developers have little experience with the term usability and, therefore, often do not consider proof-of-concept studies or validation studies to be a part of usability evaluation. This further stresses the need to provide easy access to usability evaluation information, as provided by the IUT through the Wiki page. By considering usability as a whole, WRD developers are encouraged to consider and evaluate user needs in the early technology readiness stages, which will likely increase the quality and quantity of target user involvement and thus promote UCD (Santos et al., 2021; Meyer et al., 2022).

The results from the usability evaluation confirm that the IUT supports the understanding of usability and helps to find context-specific measures. However, the selection of evaluation items was insufficiently supported. In common research practices, selecting WRD outcome measures is a prolonged process, primarily based on thorough literature search and personal experience. Through reviews of comparable state-of-the-art studies, the importance, validity, and generalizablity of evaluation items need to be carefully considered. We learned that these information points were insufficiently provided by the IUT, leading to the participants’ belief that the protocols designed with the IUT were less comparable. The Recorded Use function was intended to provide information regarding relevance and comparability on a simplified basis, using the 0–3 star rating. We learned that this rating was i) not well-understood by the participants and ii) insufficiently linked to data from actual WRD studies.

The user experiences of our study participants within the short task time strongly varied, also because different expectations about the platform were noted. By discussing these experiences and expectations at the end of the study, valuable suggestions for a new design iteration of the IUT were generated. As part of a major change, driven by the user feedback from this study, a new selection summary function was integrated in the most current, online version. It allows IUT users to get an automated analysis of the combination of their selected items. This provides better assistance with selecting measures from the recommended list, rather than leaving this entirely up to the user. Another desired, and now integrated, feature was a link to scientific literature. Shortcuts to automate search strings that contain the entered context of use were implemented to provide convenient access to relevant literature. Lastly, the database of the IUT is planned to be further expanded and refined to improve the evaluation item recommendations. The recommendations are based on Context Fit and Recorded Use, which can be improved and optimized by adding new data entries about successful applications of WRD evaluations. To accomplish this, a contribution page was launched on the website. It allows any WRD developer or researcher to register and add data based on their own work and experience.

### 5.3. Implications and importance

We believe that the optimal use of the IUT is in combination with rigorous literature research and further readings on usability evaluation practices. The individual features of the IUT are similar to clinical outcome measure databases such as the Rehab Measures Database,^5^ more general user research toolkits (Hom, 1998) or the plethora of websites that provide information on usability evaluation. Each of these resources, just like the IUT, provides users with an efficient package of information, serving as platforms for education and tools for selecting specific measures. The uniqueness of the IUT is the combination of such comparable features and the synthesis of usability information for the specific use case of wearable robotics. In addition, the dedicated, innovative study design allowed us to thoroughly evaluate, and to a certain extent validate, our platform. Our study suggests that the IUT can promote more user-oriented, mixed-method usability evaluation protocols while maintaining similar overall protocol quality. In fact, most participants pointed out that they would have liked to spend more time on the IUT to further benefit from it. However, it remains unclear what other influence the platform would have had in a more realistic time frame, and the impact on the real-life WRD applications would have to be investigated in a longitudinal follow-up study. We aim to further improve the features of the IUT, such that the use of standardized measures can be promoted to support the ongoing benchmarking endeavors of the WRD field (Torricelli et al., 2020; Crea et al., 2021). Also, individual participants highlighted that the educational and terminological endeavors should be further pushed to help create a set of global definitions and standards of usability and evaluation practices. Unified terminology would allow for more effective comparison and exchange of usability study insights, which would benefit the ultimate goal of increasing WRD acceptance.

### 5.4. Limitations

From the participants’ ratings on the SEQ and feedback in the interviews, we understand that the task of the study was inherently difficult within the set time frame. The task of defining study protocols normally is a prolonged process that involves extensive literature research and discussions with colleagues. In both conditions, the participants would have been allowed to use their traditional strategies, using any tool or information source that they would seem fit. However, due to the time pressure, most participants mainly established the protocols from their own experience and knowledge. The participants conducted very little literature research on the internet, especially in the +IUT conditions. Therefore, we have to highlight that the usability evaluation protocols designed within this study may not fully represent the usual practice. Further expanding on this point, it is not common practice to define usability studies in such a short time frame, nor defining such different protocols back-to-back. This might have introduced a learning effect, allowing experts to transfer information from their first protocol and indirectly apply it to their second; however, this limitation applied to both conditions such that the influence of the IUT could still be investigated under fair circumstances.

Furthermore, it is important to state that, especially for UC1, very little Recorded Use data had been loaded to the database at the time of the study. Therefore, the participants could not yet benefit from this feature of the IUT, which should inform them about the relevance of individual items within the greater WRD community. All data in the IUT only comprised of a sample of 158 WRD developers from our previous survey, where augmentation was represented in a small sample only (Meyer et al., 2021). Adding more data to the database will likely help better reflecting which evaluation items are used frequently in comparable WRD projects.

## 6. Conclusion

The IUT was introduced and evaluated as a platform to support WRD developers in defining usability evaluation protocols. In combination with the educational information provided, the context-specific recommendations of evaluation items positively influenced the composition of usability evaluation protocols to shift the focus toward more user-oriented, subjective assessments while maintaining the same overall quality. In the future, the IUT aims to provide a complementary option to the currently available tools to support informed decision-making when designing and conducting WRD usability studies. The IUT alone cannot improve the general practice of WRD research and development. However, our results indicated that it might be a helpful platform to support the evaluation as well as the empathizing phase of the iterative UCD process. We believe that supporting UCD endeavors will help address the residual adoption hurdles and positively impact the technology acceptance of WRD.

## Data Availability

Data and materials can be made available upon reasonable request to the authors.

## Footnotes

1
https://www.sralab.org/rehabilitation-measures.

2
https://www.safearoundrobots.com/.

3
https://www.sralab.org/rehabilitation-measures.

4
https://www.physio-pedia.com/home/.

5
https://www.sralab.org/rehabilitation-measures.

## Supplementary material

For supplementary material accompanying this paper visit https://doi.org/10.1017/wtc.2022.31. click here to view supplementary material
