# Supplementary material for: Design and validation of a novel online platform to support the usability evaluation of wearable robotic devices
Source: Wearable Technol. 2023 Jan 24;4:e3. doi: 10.1017/wtc.2022.31 (PMC10936320; doi:10.1017/wtc.2022.31)
Supplement: Supplementary file 1 [file S2631717622000317sup001.pdf]

Protocol Evaluation Sheet

Overall, how would you rate the quality of the evaluation protocol?	Very Poor									Very Good
	1	2	3	4	5	6	7	8	9	10

Please indicate your level of agreement with the following statements:	Strongly Disagre	Disagre	Neutral	Agree	Strongly Agree	Comments
An appropriate **study aim** was defined						
An appropriate **study participant group** was defined						
An appropriate form of **target (end)user involvement** was defined						
An appropriate **selection of evaluation measures** was defined						
The proposed study is **feasible to conduct**						
The proposed study **contains references** to benchmarks or studies						
The proposed study would **generate meaningful design insights**						
The proposed study would **yield generalizable results**						

How would you categorise the focus of the proposed study protocol?

Please allocate a total of 100 points to the three usability dimensions: effectiveness, satisfaction and efficiency

Effectiveness	0
Satisfaction	0
Efficiency	0
Total (must be 100)	0
